# Complete chloroplast genome of Chilean needle grass, *Nassella neesiana* (Poaceae: Stipeae)

**DOI:** 10.1080/23802359.2017.1390414

**Published:** 2017-10-17

**Authors:** Aisuo Wang, Hanwen Wu, David Gopurenko

**Affiliations:** a NSW Department of Primary Industries, Wagga Wagga Agricultural Institute, PMB, Wagga Wagga, Australia;; b Graham Centre for Agricultural Innovation, Wagga Wagga, Australia

**Keywords:** Illumina sequencing, stipoid, invasive weed

## Abstract

*Nassella neesiana* (Chilean needle grass) is a serious weed in Australia, and has been included in the list of Weeds of National Significance (WoNS). We present here, the complete chloroplast sequence of *N. neesiana* reconstructed from Illumina whole genome sequencing. The complete chloroplast sequence is 137,700 bp in size, and has similar gene content and structure as other published chloroplast genomes of Stipeae. The *N. neesiana* chloroplast genome is deposited at GenBank under accession number MF480752.

*Nassella neesiana* (Trin. & Rupr.) Barkworth (Poaceae: Stipeae), commonly known as Chilean needle grass, is one of the Weeds of National Significance (WoNS) in Australia (CRC 2003). It displaces palatable grasses from pastures, decreases productivity of grazing livestock, and significantly degrades biodiversity in native grasslands. Development of genetic diagnostics to distinguish *N. neesiana* from similar appearing native grasses is essential to the effective management of this weed. Earlier attempts at genetic diagnostics of *N. neesiana* have been generally hindered by a lack of informative loci to adequately distinguish it from several other stipoid grass species (Wang et al. 2014; Wang et al. 2017). Provision of genomic data from *N. neesiana* is required for identification of novel informative loci that could be used in routine genetic diagnostic approaches such as DNA barcoding and Loop mediated isothermal amplification.

We assembled the complete chloroplast of *N. neesiana* after Illumina sequencing of a specimen collected from Wagga Wagga NSW in Australia. The specimen was taxonomically identified as *N. neesiana* and retained at Wagga Wagga Agricultural Institute (WWAI) under voucher number ww19860.

Total genomic DNA was extracted from fresh leaves of the specimen using a modified CTAB protocol (Wang et al. 2016). A full lane of PE125 Illumina sequencing based on a 500 bp library was conducted by BGI (Hongkong). NOVOPlasty (Dierckxsens et al. 2017) was applied to extract chloroplast genome sequences from the raw reads of the fastq files (14,276,252 reads for each direction). The recommended K-mer value (39) was applied, and the chloroplast genome of *Brachyelytrum aristosum* (NC_027470.1) was used as a seed input in the analysis.

Annotation of the *N. neesiana* chloroplast genome was performed using both CpGAVAS (Liu et al. 2012) and DOGMA (Wyman et al. 2004) with default settings. The predicted annotations were verified using BLAST search (Altschul et al. 1990).

All complete chloroplast sequences for the tribe Stipeae were downloaded from GenBank, including *Stipa hymenoides* (NC 027464), *Oryzopsis asperifolia* (NC 027479), *Piptochaetium avenaceum* (NC 027483), *Stipa lipskyi* (NC 028444), and *Stipa purpurea* (NC 029390.1). These sequences were aligned with the corresponding sequences of *N. neesiana* and one outgroup (*Triticum turgidum*, NC_024814.1) using MAFFT (Katoh and Standley 2013) at the default settings. The resulting alignment file was applied to construct a Maximum Likelihood (ML) tree under the GTR nucleotide substitution model (plus Gamma distribution) in PhyML 3.1 (Guindon et al. 2009) (Figure 1). The analysis was tested at 1000 Bootstrap replications.

**Figure 1.**
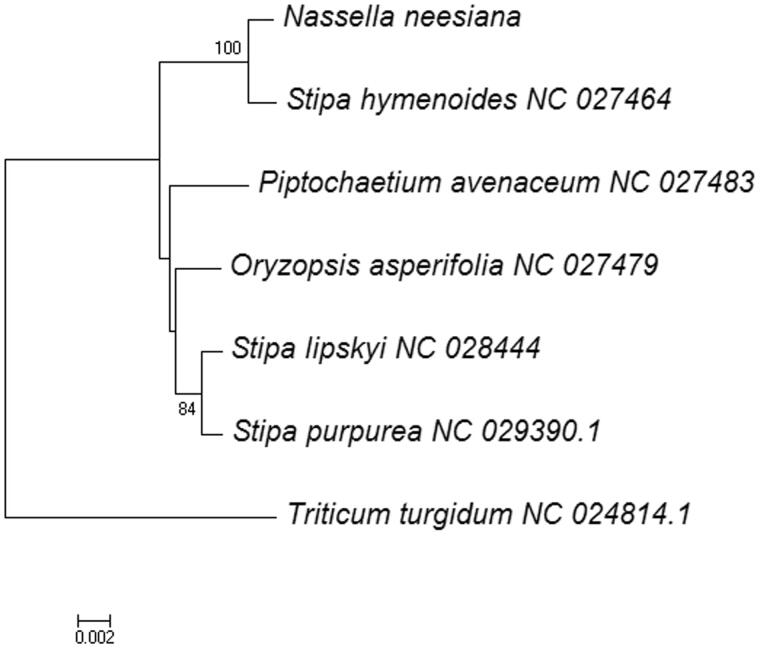
Phylogenetic tree produced using Maximum Likelihood of complete chloroplast genomes from tribe Stipeae with *Triticum turgidum* as the outgroup.

The complete chloroplast sequence of *N. neesiana* is 137,700 bp. The gene content, gene order and structure of the inverted repeats are similar to most members of the tribe except for *Stipa lipskyi*. The ML phylogenetic tree of tribe Stipeae, constructed on the complete chloroplast sequences, groups *N. neesiana* together with *Stipa hymenoides* with strong bootstrapping support (100). The complete chloroplast genome of *N. neesiana* (GenBank accession MF480752) provides a platform by which further genetics studies (such as DNA barcoding, evolution and phylogeny of *Nassella* species) can be conducted, thereby contributing to the management of this weed.
